# Correction: Association of electronic learning devices and online learning properties with work-related musculoskeletal disorders (WMSDs): A cross-sectional study among Thai undergraduate students

**DOI:** 10.1371/journal.pone.0327055

**Published:** 2025-06-25

**Authors:** Thanyaporn Direksunthorn, Panicha Polpanadham, Ueamporn Summart, Khannistha Mahem, Pipatpong Kempanya, Muhamad Zulfatul A’la, Yuwadee Wittayapun

In the Ethical approval statement, the ethical approval reference number from the Walailak University Institutional Review Board is listed incorrectly. The correct ethical approval reference number is: WUEC-22-077-01.
