# Supplementary material for: Association of electronic learning devices and online learning properties with work-related musculoskeletal disorders (WMSDs): A cross-sectional study among Thai undergraduate students
Source: PLoS One. 2023 Oct 30;18(10):e0291597. doi: 10.1371/journal.pone.0291597 (PMC10615310; doi:10.1371/journal.pone.0291597)
Supplement: Attachment — Submitted filename: Response to Reviewers.docx [file pone.0291597.s002.docx]

**Journal Requirements: Response**

1. Please ensure that your manuscript meets PLOS ONE's style requirements, including those for file naming.

**Author’s Response:** I have made corrections to ensure that our manuscript confirms the PLOS ONE's style requirements.

2. Please provide additional details regarding participant consent. In the ethics statement in the Methods and online submission information, please ensure that you have specified what type you obtained (for instance, written or verbal, and if verbal, how it was documented and witnessed). If your study included minors, state whether you obtained consent from parents or guardians. If the need for consent was waived by the ethics committee, please include this information.

**Author’s Response:** We already stated, “All students who consented to participate in the study checked the "**I accept to participate**" checkbox online before the survey started as part of the recruitment of participants. We did not include minors in this study.

3. Thank you for stating the following financial disclosure:

“NO - Include this sentence at the end of your statement: The funders had no role in study design, data collection and analysis, decision to publish, or preparation of the manuscript”

We have already removed funding information that appeared in the Acknowledgments and Funding section and any other section of our manuscript. However, we added the information in our cover letter.

a)        Please clarify the sources of funding (financial or material support) for your study. List the grants or organizations that supported your study, including funding received from your institution.

**Author’s Response:** We included our amended statement in our cover letter that “The study received financial support from Walailak University through the "Walailak University's Individual Research Grants" (Grant Number WU-IRG-65-015).

b)        State what role the funders took in the study. If the funders had no role in your study, please state: “The funders had no role in study design, data collection and analysis, decision to publish, or preparation of the manuscript.”

**Author’s Response:** The funders had no role in study design, data collection and analysis, the decision to publish, or the preparation of the manuscript. (Please see the source of internal research grants via the website URL: https://riie.wu.ac.th/?page_id=5876.)

c)        If any authors received a salary from any of your funders, please state which authors and which funders.

**Author’s Response:** None of authors received salary from funder.

d)        If you did not receive any funding for this study, please state: “The authors received no specific funding for this work.”

**Author’s Response:** Not applicable

Please include your amended statements within your cover letter; we will change the online submission form on your behalf.

**Author’s Response:** We included our amended statements within our cover letter.

4. In your Data Availability statement, you have not specified where the minimal data set underlying the results described in your manuscript can be found. PLOS defines a study's minimal data set as the underlying data used to reach the conclusions drawn in the manuscript and any additional data required to replicate the reported study findings in their entirety. All PLOS journals require that the minimal data set be made fully available. For more information about our data policy, please see <http://journals.plos.org/plosone/s/data-availability>.

Upon re-submitting your revised manuscript, please upload your study’s minimal underlying data set as either Supporting Information files or to a stable, public repository and include the relevant URLs, DOIs, or accession numbers within your revised cover letter. For a list of acceptable repositories, please see <http://journals.plos.org/plosone/s/data-availability#loc-recommended-repositories>. Any potentially identifying patient information must be fully anonymized.

**Author’s Response:** We have already generated our minimal data set as a supporting information file as (S1.XLSX).

We will update your Data Availability statement to reflect the information you provide in your cover letter.

**Author’s Response:** Thank you.

5. We note that you have stated that you will provide repository information for your data at acceptance. Should your manuscript be accepted for publication, we will hold it until you provide the relevant accession numbers or DOIs necessary to access your data. If you wish to make changes to your Data Availability statement, please describe these changes in your cover letter and we will update your Data Availability statement to reflect the information you provide.

**Author’s Response:** We have already produced the minimal data set. (S1.XLSX)

**Reviewers' comments: Reviewer #1**

1. General Comments
? Why electronic learning? Is that only during the era of COVID 19? It’s known that electronic learning induces Musculo-skeletal disorders. Hence, what is new? What is new among university students? When did actual Musculo-skeletal formation end?

**Author’s Response:** We clarified these concerns in the introduction section as.

Electronic learning has been introduced in the adverse situation of COVID19 lockdown to bring the students in a new-normal lifestyle and get them triggered against the challenges.

Although the impact of electronic learning in the induction of Musculo-skeletal disorders is literally known, the very particular effect in Thailand is reflected for the first time through this study. Additionally, the nature of disadvantages that cause postural disorder and eventually Musculo-skeletal disorders of the university students has also been unraveled for the Thai students by this study.

Formation of Musculo-skeletal disorders were reported be ended through the well-off economic condition, consciousness of healthy lifestyle, and the knowledge on the postural balance which together affirmed the improvement of the situation.

? Why cross sectional? Why not other design?

**Author’s Response:** We appreciate the thoughtful question the reviewer. In fact, this study was a component of a larger research project titled “**Effects of e-learning during the COVID-19 pandemic on the prevalence and factors associated with musculoskeletal disorders (MSDs) among Thai, Indonesian, Vietnamese, and Laos faculty members and students**”. You may be agreed that cross-sectional studies analyze data from a population at a point in time, and this study was designed to conduct in a particular time-point from April to June 2022 during the countrywide lockdown and social gathering prohibition due to the coronavirus outbreak.

Other designs such as longitudinal study or experimental study does not fit with our research problem and research question.

What is the final sampling procedure used to reach the study participants?

**Author’s Response:** Very valuable question indeed. Simple Random Sampling (SRS) procedure was adopted as the final sampling procedure.

2. Specific Comments
• All the sections of the manuscript are weak and needs strengthening.

**Author’s Response:** We have rigorously revised the manuscript and strengthened all possible weakness and errors throughout the manuscript.

• Try to present as per the PLOS ONE standard/ Journal format/

**Author’s Response:** We checked the format to make compatible with PLOS ONE.

• The knowledge gap is described inadequately.

**Author’s Response:** Thank you very much for your insightful comment. However, we have meticulously reviewed the manuscript and tried to enumerate the research gap of the study. The horror of the pandemic, its devastating role in deadliness, and its way out to mobilize the young generation through electronic learning were embarked on as part of the research question. However, the negative effect of positional and postural imbalance has been analyzed very critically incorporating other socioeconomic conditions as etiology. The factual differences between this COVID19 pandemic with other outbreaks have been considered. How the effect of electronic learning may be equally disrupting muscle and muscular health in emerging and reemerging infectious diseases leading to pandemics.

The study also tried to emphasize that physical learning compared to electronic learning speculating not only the MSDs but also other probable physical and mental disorders. Additionally, psychosocial factors along with physical factors have pertained as the primary risk factors. The effect of electronic learning on pain intensity is also measured which is usually ignored in Nordic questionnaire assessment system. The existing literature failed to incorporate the characterization of pain levels and risk perception to ensure a comprehensive approach to capturing work-related MSDs (WMSDs), this research unveiled the setbacks of related research.

• The methods are also inadequately described and ensure the completeness of all its sub-sections.

**Author’s Response:** Thank you so much for your observation. The authors have rigorously revised the methods to ensure the completeness of all of the subsections

• How and when was data quality assurance was ensured?

**Author’s Response:** We appreciate your question. We ensured data quality through various steps, including content validity assessment, pilot testing, meetings with research partners, data cleaning, and verification of basic statistical assumptions. The details of the validation approach have been contextualized in the manuscript through:

A. index of item objective congruence (IOC) by three experts, whose IOCs ranged from 0.67 to 1.00.

B. The internal consistency of the stress scale using Cronbach's alpha coefficient.

C. Researchers-participants collaboration

D. Participant’s Feedback from QR code; and

E. Data cleaning to check the response consistency and exclude cases not meeting the eligibility criteria.

‘• Use of language e.g., multi-variate analysis needs revision.

**Author’s Response:** We improved the quality of the text by utilizing English-language editing services from Editage (www.editage.com).

• The results didn’t report confidence interval?

**Author’s Response:** We reported the confidence interval in Table 3-4.

How was the case to variable ratio calculated?

**Author’s Response:** Thank you so much for your inquisition. The issue is detailed in the manuscript to highlight the importance of a higher case-to-variable ratio.

According to the simplest rules of cases-to-IVs for logistic analysis planned for use, the number of cases should be greater than 50 + 8 m, where “m” represents the number of independent variables (IVs) [22]. Thirteen IVs were used in this study. Hence, 4,618 cases exceeded the threshold of 154.

Is the model fitted? How?

**Author’s Response:** This is quite a befitting model and findings (constant of -2.776) showed that the data fitted with the logistic model (p = 0.164); the classification accuracy of the risk prediction model was 64.7%.

• Ensure the completeness of all sections E.g. The result and the discussion sections. • The key terms are incomplete and the reference needs meticulous revision.

**Author’s Response:** We carefully reviewed the revised manuscript to change the structure and clarity of the text to ensure that the data and discussion were presented clearly and objectively. We made revisions to the reference to ensure its accuracy. Nonetheless, we have rigorously revised all other sections to make each section complete and significantly meaningful.

**Reviewers' comments:** Reviewer #2

i) A professional proof reading is recommended for typos and grammatical errors

**Author’s Response:** We improved the quality of the text with the help of professional English-language editing services - Editage (www.editage.com).

ii) the sampling technique needs justification

**Author’s Response:** Thank you so much for your valuable suggestion for improvement. The sampling technique is clarified as:

Based on the 2021 Thailand Nursing and Midwifery Council database [23], 96 nursing institutes were distributed across five regions. Using multistage sampling, two of the five regions were selected in the first step, namely the southern and northeastern regions. In these regions, there are 37 nursing institutes with three affiliations, including the Ministry of Education, the Ministry of Public Health, and the Private Sector. Fifteen nursing institutions were selected using a non-proportional stratified sampling technique. In addition, three nursing faculties were conveniently sampled from the central region, reaching a total of 18 faculties. The 18 institutions sampled had 8,534 students. Research partners at the 18 institutions were able to provide an information sheet and a method for providing an online voluntary consent form by clicking on a voluntary response box for 5,395 students. Of the 5,395 students, 5,136 1st–4th-year students indicated a willingness to voluntarily participate in this study. Code numbers were created to safeguard students’ privacy. Of the 5,136 students, simple random sampling was performed to sample 4,618 1st–4th-year undergraduate students (Fig 1). Only the authors of this study had access to individual students’ data.

iii) the limitations of the study need to be clearly highlighted/acknowledged in the discussion component

**Author’s Response:** The limitations of the study are approached at the end of the discussion section. The prospect and thumb message of the study is clearly delineated in the light of the discussion component.
